# Comparison of hypnosis plus sedoanalgesia and sedoanalgesia alone methods used in the ERCP procedure: A prospective randomized study

**DOI:** 10.1097/MD.0000000000042641

**Published:** 2025-06-06

**Authors:** Ufuk Arslan, Şeyda Efsun Özgünay, Direnç Yiğit, Yiğit Düzköylü

**Affiliations:** a Department of Gastroenterological Surgery, Bursa NEV Hospital, Bursa, Turkey; b Department of Anesthesiology and Reanimation, Yuksek Ihtisas Teaching and Research Hospital, Bursa, Turkey; c Department of Gastroenterological Surgery, Antalya OFM Hospital, Antalya, Turkey; d Department of Gastroenterological Surgery, Basaksehir Cam Sakura City Hospital, Istanbul, Turkey.

**Keywords:** endoscopic retrograde cholangiopancreatography, hypnosis, outpatient anesthesia, sedation analgesia

## Abstract

Endoscopic retrograde cholangiopancreatography (ERCP) is an interventional procedure that is often performed under sedation anesthesia and that is used in the diagnosis and treatment of hepatopancreaticobiliary diseases. The objective of this study was to compare the efficacy of hypnosis in conjunction with sedoanalgesia and sedoanalgesia alone in the context of outpatient anesthesia prior to ERCP. Patients undergoing ERCP in the endoscopy unit between March and May 2021 were randomly assigned to 1 of 2 groups: group 1 received hypnosis and sedoanalgesia, and group 2 received sedoanalgesia alone. Both groups were administered 0.5 to 0.6 mg/kg intravenous pethidine hydrochloride (HCl), 1 to 3 mg intravenous midazolam, and 1 to 2 mg/kg intravenous propofol. The first group also received hypnotic induction before the procedure and anesthetic agents. In the event of patients exhibiting movement during the procedure, an anesthesiologist was unaware of the patient’s allocation and administered additional propofol and/or pethidine HCl. A statistical comparison was conducted between the 2 groups regarding demographic data, vital parameters, medication requirements, and satisfaction scales. Thirty patients were included in both groups. Following the procedure, the administration of propofol and pethidine HCl was reduced in group 1 (*P* = .031 and *P* = .009, respectively). The 5-minute heart rate, baseline peripheral oxygen saturation at 3 and 10 minutes were lower in group 2 (*P* = .008, *P* = .011, *P* = .017, and *P* = .031, respectively). Although the dose of anesthetic drugs were lower, no significant difference was observed neither in the patient satisfaction scores, nor in patient movements. The use of hypnosis during ERCP enhances the efficacy of sedoanalgesia. Hypnotic anesthesia may be employed as an alternative method in cases where high-dose administration of these agents is contraindicated.

## 1. Introduction

Endoscopic retrograde cholangiopancreatography (ERCP) is an endoscopic procedure typically performed under outpatient sedation for the diagnosis and treatment of liver, biliary, and pancreatic diseases.^[1]^ For procedural anesthesia, opioid analgesics such as pethidine hydrochloride (HCl) are commonly combined with benzodiazepines such as midazolam and intravenous anesthetic agents such as propofol and ketamine HCl are also used.^[1–3]^ Despite the enhanced comfort of the patient and the endoscopist during the procedure, the use of these agents necessitates meticulous post-procedural monitoring, resulting in prolonged awakening and return to home.^[4]^ The underlying cause of this prolonged awakening was the use of pharmacological agents. Furthermore, the use of these agents may result in adverse effects, including allergies, respiratory depression, hemodynamic changes, and cardiac arrhythmia.^[5]^

In endoscopic procedures, hypnosis can be employed as an alternative method to provide comparable levels of comfort while reducing the incidence of side effects. Hypnosis has been shown to reduce pain perception by elevating the pain threshold.^[6–8]^ It has been shown to be an effective treatment for chronic, acute and postoperative pain. Owing to its noninvasive nature, this technique is advantageous in achieving cooperation and reducing anxiety and pain in patients undergoing anesthesia. Furthermore, it has the advantage of enabling the patient to awaken promptly following the procedure and to return home rapidly.^[7,9]^ Hypnosis employs techniques that target the patient’s sensory-cognitive state, physiological changes and specific symptoms.^[10–12]^ Hypnosis is an effective treatment for pain and anxiety, when used as an adjuvant in surgical patient.^[13–15]^

This study aimed to compare the efficacy of sedoanalgesia, a method of outpatient anesthesia administered before ERCP, and adjuvant hypnosis added to sedoanalgesia in terms of medication consumption, hemodynamic stability, oxygen saturation, and patient satisfaction.

## 2. Materials and methods

### 2.1. Study design

Sixty patients undergoing therapeutic ERCP with outpatient anesthesia in our endoscopy unit between March and May 2021 were randomly assigned to 2 groups using the sealed envelope technique. Patient data were recorded and evaluated prospectively. Prior to the commencement of the study, each patient was provided with comprehensive information and asked to provide written informed consent. This study was conducted in accordance with the principles set forth in the Declaration of Helsinki of the World Medical Association. This study was approved by the Ethics Committee of the Turkish Republic Ministry of Health General Directorate of Health Services (No. 77979112) on 01/02/2021.

In our clinic, we perform 36 ± 4 ERCP procedures per month. Following patient inclusion criterion, 60 patients were found to be suitable for the study. Patient selection was based on the presence of elevated cholestatic enzymes or intra- or extrahepatic bile ducts, Wirsung duct, and periampullary pathologies as identified by magnetic resonance cholangiopancreatography. These patients were referred for therapeutic endoscopic retrograde cholangiopancreatography (ERCP). Patients scheduled for ERCP, between the ages of 18 and 80 years, who were eligible for anesthesia and consented to hypnosis were included in the study. Patients who did not wish to be hypnotized, did not consent to participate in the study, could not be administered anesthesia, and scored below 4 on the Tastan Hypnotic Suggestibility Scale were excluded.^[16]^

### 2.2. The study procedure

The study procedure entailed the performance of ERCP by the same endoscopist and the administration of hypnosis procedures by the same anesthesiologist. The Pentax® ED34-i10T Video Duodenoscope was used in the ERCP procedures. Prior to the procedure, all patients were provided with an anesthesia consultation. Should necessity arise, patients were referred for consultation with other departments, such as cardiology and pulmonary diseases. Prior to the commencement of the procedures, patients were required to abstain from food and drink for a minimum of 8 hours. For patients taking anticoagulant medication, this was discontinued for a period of 1 week prior to the procedure. Prophylactic low-molecular-weight heparin was administered if deemed necessary. All procedures were performed in the prone position. In all patients, anesthesia was topically administered to the throat using 10% lidocaine. The contrast agent used was Urografin® diluted 1:3.

The patients recruited for the study were randomly assigned to 1 of 2 groups: group 1, which received sedoanalgesia and hypnosis, and group 2, which received only sedoanalgesia. Both groups were administered 1 to 3 mg/kg intravenous midazolam, 1 to 2 mg/kg intravenous propofol and 0.5 to 0.6 mg/kg intravenous pethidine HCl, in accordance with standard practice at the outset of the procedure. The initial cohort additionally underwent hypnosis before the procedure. In cases where patients exhibited movement and a mean arterial pressure (MAP) of 20% above baseline, a blinded anesthesiologist administered propofol and/or pethidine HCl. The following parameters were recorded a baseline and at 3, 5, and 10 minutes including heart rate, oxygen saturation (SpO_2_), and MAP. A statistical comparison was conducted between the 2 groups with regard to demographic data, ERCP data (including the duration of the procedure, success of cannulation, and incidence of complications), vital parameters, medication requirements, and satisfaction scales. A flowchart is shown methodology.

### 2.3. The method of hypnosis

The method was explained to the patient, who was informed that it could be used for relaxation and pain relief. Prior to the patient’s transfer to the intervention room, hypnotic induction was administered in the premedication room via visual identification and verbal suggestions. Subsequently, progressive muscular relaxation was performed.^[17,18]^ Following relaxation of the extremities, trunk, head, neck, and whole body in accordance with hypnotic induction, suggestions were provided. The patient was prompted to imagine a mountain hut and snowy landscape and to construct a snowball outside, experiencing the cold and resulting numbness in the hands. It was indicated that the subject wore a glove that had the capacity to induce a numbing and cold sensation up to the wrist. The patients were instructed to open their eyes and observe that they did not experience pain when the hand was pricked with a needle. Subsequently, the patient was presented with a metaphor that this sensation was akin to consuming ice cream. Cold ice cream is conceptualized as passing through the throat and spreading to the esophagus, stomach, and intestines.^[19]^ It was stated that the numbness would dissipate in the awakening room but that the feeling of comfort would persist until the patient’s departure from the hospital. The intervention commenced with the patient being instructed to take a deep breath through their nose when they felt ready. They were then asked to fill their lungs fully and then to relax by slowly exhaling through the mouth, allowing any concerns to dissipate. Subsequently, an anesthetic protocol was initiated.

### 2.4. Statistical analysis

The Shapiro–Wilk test was employed to ascertain the normality assumption for continuous variables. In accordance with the findings of the normality test, continuous and discrete study variables are expressed as median (minimum: maximum) and mean ± standard deviation, respectively. Categorical variables are expressed as numbers and percentages. To compare the study groups, the independent samples *t* test, Mann–Whitney *U* test, chi-square test, and Fisher exact test were employed. Statistical significance was set at *P* < .05. The data were analyzed using the statistical software package SPSS (IBM Corp.). Published in 2015. The IBM SPSS Statistics software package (IBM Corp., Armonk, NY) was used for statistical analyses, with a type I error rate of 5% for statistical comparisons.

## 3. Results

As illustrated in Table 1, the median age of the hypnosis group was 58 years (range: 25–77 years), while that of the sedation group was 50 years (range: 23–73 years). The mean age of the hypnosis group was 52.3 years, while that of the sedation group was 50.4 years. No statistically significant difference in age was observed between the 2 groups (*P* = .668). The mean body mass index (BMI) was 26.93 ± 4.38 kg/m² in the hypnosis group and 25.90 ± 4.28 kg/m² in the sedation group, indicating that there was no significant difference in BMI between the 2 groups (*P* = .361). The distribution of American Society of Anesthesiologists scores was similar between the 2 study groups (*P* = .168). The prevalence of comorbidities was 40% in the hypnosis group and 46.7% in the sedation group, indicating that the incidence of comorbidities did not differ significantly between the 2 groups (*P* = .602).

**Table 1:** Comparison of demographic characteristics between the groups.

	Hypnosis (n = 30)	Sedation (n = 30)	*P*-value
Age (yr)	58 (25:77)	50 (23:73)	.668*
Gender (F/M)	16/14	18/12	.602†
BMI	26.93 ± 4.38	25.90 ± 4.28	.361‡
ASA			
I	11 (36.70%)	15 (50%)	.168†
II	15 (50%)	8 (26.70%)	
III	4 (13.30%)	7 (2.30%)	
Comorbidity	12 (40%)	14 (46.70%)	.602†

Data were presented as median (minimum: maximum), mean ± standard deviation, and n (%).

ASA = American Society of Anesthesiologists, BMI = body mass index.

* Mann–Whitney *U* test.

† Chi-square test.

‡ Independent samples *t* test.

As shown in Table 2, there was no statistically significant difference in the duration of the procedure between the study groups (*P* = .666). Furthermore, the rates of cannulation (90% vs 90%) and complications (3.30% vs 6.70%) did not differ between the groups (*P* > .99 and *P* > .99, respectively). Furthermore, no significant difference was observed in the patient satisfaction scores between the hypnosis and sedation groups (*P* = .770). The median satisfaction score was 9 (range, 5–10) in the hypnosis group and 9 (range 6–10) in the sedation group. The median score on the hypnosis scale for the hypnosis group alone was 5 (range 4–7). The mean satisfaction scores for the hypnosis group was 8.5, while the mean satisfaction score for the sedation group were 8.7. The mean score on the hypnosis scale in the hypnosis group was 4.8. The second group was not subjected to hypnosis. The rate of dormicum administration did not differ between the groups (*P* = .426). Conversely, there was a notable discrepancy in the administration of propofol and pethidine HCl between the 2 groups 44. The administered doses were higher in the sedation group. The number of patient movements was similar between the groups (*P* = .771).

**Table 2:** Comparison of procedural data between the groups.

	Hypnosis (n = 30)	Sedation (n = 30)	*P*-value
Duration of procedure (min)	9 (6:17)	9.50 (6:19)	.666*
Cannulation	27 (90%)	27 (90%)	>.99‡
Complications	1 (3.30%)	2 (6.70%)	>.99‡
Patient satisfaction	9 (5:10)	9 (6:10)	.770*
Hypnosis scale	5 (4:7)	NA	NA
Midazolam			
1 mg/kg	9 (30%)	7 (23.30%)	
2 mg/kg	17 (56.70%)	15 (50%)	.426†
3 mg/kg	4 (13.30%)	8 (26.70%)	
Propofol (mg)	55 (50:100)	60 (50:120)	**.031** *
Pethidine HCl (mg)	30 (25:50) 30.83 *±* *5.74*	30 (25:50) 34.83 *±* *7.01*	**.009** *
Patient mobilization	1 (0:3)	1 (0:3)	.771*

Data were presented as median (minimum: maximum), mean ± standard deviation, and n (%). Bold values indicate statistically significant.

NA = not available.

* Mann–Whitney *U* test.

† Chi-square test.

‡ Fisher exact test.

**Figure 1.**
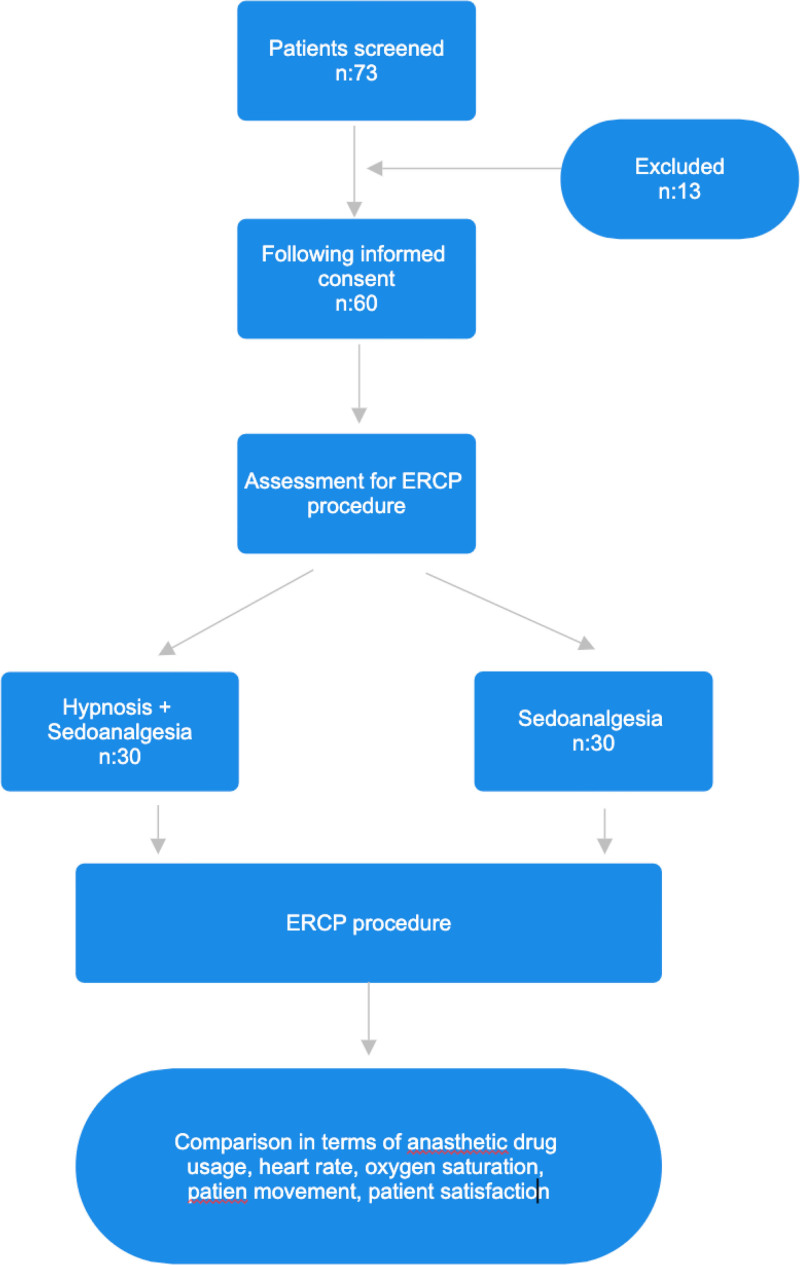
Flowchart of patient selection and study design.

As illustrated in Table 3, no significant difference was observed in the baseline and 3-minute heart rate measurements between the groups (*P* = .476 and *P* = .559, respectively). Conversely, the mean 5-minute heart rate was higher in the hypnosis group (*P* = .008). Furthermore, no significant difference was observed in the 10-minute heart rate measurements between the 2 groups (*P* = .824). Upon examination of the percentage change in measurements from baseline to minutes 3, 5, and 10, it was observed that there was an overall increase in the hypnosis group compared with the sedation group. However, these changes were not statistically significant (*P* > .05) (Fig. 1).

**Table 3:** Comparison of vital parameters and recovery score between the groups.

	n	Hypnosis	n	Sedation	*P*-value
Heart rate					
Baseline	30	85.20 ± 2.89	30	85.23 ± 16.15	.476†
3-Minute	30	84.50 (57:121)	30	84.50 (57:121)	.559†
5-Minute	30	91.20 ± 19.77	30	79.20 ± 13.75	**.008** *
10-Minute	13	82.08 ± 15.90	15	80.60 ± 18.57	.824*
Δ%_3-Minute→baseline_	30	↑2.42 (‐22.89:77.97)	30	↓8.73 (‐30.43:62.07)	.587*
Δ%_5-Minute→baseline_	30	↑6.79 (‐42.55:145.76)	30	↓2.17 (‐46.15:68.97)	.139*
Δ%_10-Minute→baseline_	13	↑1.28 (‐32.41:86.44)	15	↓13.54 (‐40.18:41.07)	.717*
Mean arterial pressure					
Baseline	30	89.50 (72:115)	30	85 (73:117)	.599*
3-Minute	30	92.47 ± 12.47	30	88.40 ± 11.58	.196†
5-Minute	30	95.90 ± 14.97	30	92.30 ± 10.96	.292†
10-Minute	13	89.92 ± 9.21	15	86.67 ± 9.76	.373†
Δ%_3-Minute→baseline_	30	↑4.93 ± 21	30	↑1.29 ± 12.88	.421†
Δ%_5-Minute→baseline_	30	↑9.17 ± 24.56	30	↑6.86 ± 19.65	.421†
Δ%_10-Minute→baseline_	13	↑4.76 (‐12.50:10.84)	15	↑1.35 (‐28.57:30.38)	.650*
Peripheral oxygen saturation					
Baseline	30	98 (92:100)	30	96.50 (88:100)	**.011** *
3-Minute	30	97.50 (91:100)	30	95 (88:100)	**.017** *
5-Minute	30	95.60 ± 3.57	30	94.53 ± 3.49	.247†
10-Minute	13	96.85 ± 2.67	13	94.80 ± 2.46	**.031** †
Δ%_3-Minute→baseline_	30	↓1.11 ± 3.55	30	↓1.18 ± 4.96	.955†
Δ%_5-Minute→baseline_	30	↓2.26 ± 3.65	30	↓1.42 ± 4.33	.421†
Δ%_10-Minute→baseline_	13	↓0.68 ± 3.88	13	↓1.37 ± 3.88	.588†
MAS					
Baseline	30	8 (7:10)	30	8 (7:10)	.428*
5-Minute	30	10 (9:10)	30	10 (9:10)	.563*
10-Minute	30	10 (10:10)	30	10 (10:10)	>.99*
Δ_5-Minute–baseline_	30	1 (0:3)	30	1.50 (0:30)	.611*
Δ_10-Minute–baseline_	30	2 (0:3)	30	2 (0:3)	.428*

Data were presented as median (minimum: maximum) and mean ± standard deviation, and n (%). Δ%3-Minute → baseline percentage change in measurement from admission to minute 3. Δ%5-Minute → baseline percentage change in measurement from admission to minute 5. Δ%10-Minute → baseline percentage change in measurement from admission to minute 10. Δ5-Minute – baseline: subtraction of the admission score from the 5-Minute score. Δ10-Minute – baseline: subtraction of the admission score from the 10-Minute score. Bold values indicate statistically significant.

MAS = Modified Aldrete Score.

* Mann–Whitney *U* test.

† Independent samples *t* test.

Upon examination according to MAP measurements, no discernible difference was observed between the 2 groups at the time of admission or at the subsequent 3-, 5-, and10-minute intervals. Furthermore, analysis of the change in related measurements from admission revealed no statistically significant difference between the 2 groups (Fig. 1). Comparisons of SpO_2_ measurements revealed that the median saturation levels measured at admission (98 vs 96.50) and at minute 3 (97.50 vs 95) were higher in the hypnosis group (*P* = .011 and *P* = .017, respectively). Nevertheless, no significant difference was observed in the 5-minute saturation measurements between the 2 groups (*P* = .247). Examination of the 10-minute saturation measurements (96.85 vs 94.80) revealed that the mean saturation level was also higher in the hypnosis group (*P* = .031). Nevertheless, no significant difference was observed in the percentage change in saturation levels from admission to minutes 3, 5, and 10 between the 2 groups (*P* > .05) (Fig. 1). The mean values for the hypnosis group were 97.8, 96.7, 95.6 and 96.8 for peripheral oxygen saturation (POS) at baseline, POS 3, POS 5, and POS 10 minutes, respectively. The mean values in the sedation group were 95.9, 97.4, 94.5 and 94.8 for POS at baseline, 3, 5, and 10 minutes, respectively.

**Figure 2.**
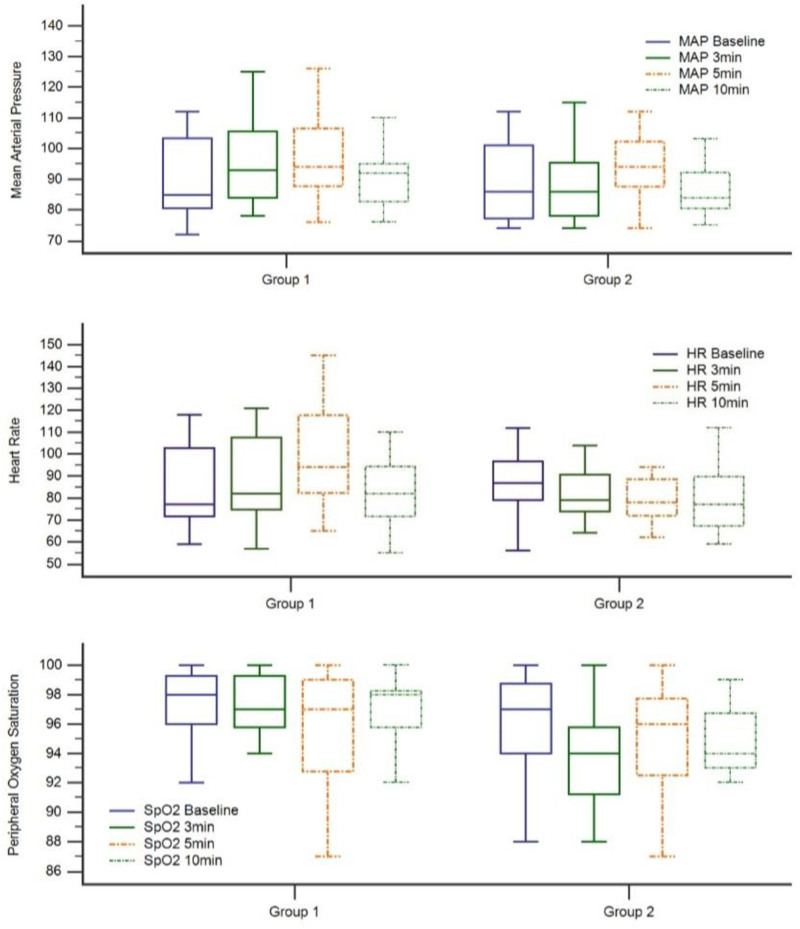
Graph of vital parameter distribution by groups.

The modified Aldrete score revealed no statistically significant difference in admission, 5-minute, and 10-minute scores between the 2 groups. Furthermore, the calculated “score of difference” values, obtained by subtracting the admission score from the 5-minute and 10-minute scores, did not differ between the groups (Fig. 2).

## 4. Discussion

In our prospective clinical study, we aimed to evaluate the efficacy of hypnosis and its advantage when it is used in conjunction with standard analgesia in endoscopy in terms of anesthetic dosing, haemodynamic findings and overall patient satisfaction. Endoscopic interventions are procedures employed for the diagnosis and treatment of gastrointestinal diseases in patients of all ages. During these procedures, sedative anesthesia is frequently employed to enhance patient and physician comfort and augment the success of the procedure.^[20]^ Despite the varying administration and dosage of anesthesia contingent on several parameters, including age, BMI, comorbidities, and medications used, none of the patient groups experienced the same effects.^[21]^ Anesthesia administration can be challenging in patient groups with a high number of comorbidities, particularly due to nutritional disorders, impaired laboratory parameters and primary diseases. Furthermore, patients who have received anesthetic agents require close monitoring and follow-up following the procedure. This ultimately results in a loss of workforce for healthcare workers and delayed return to work for patients. These agents have the potential to cause respiratory depression and cardiac side effects as well as hepatotoxic and nephrotoxic side effects.^[22]^ In light of these considerations, anesthesia with hypnosis, which is one of the methods that allows for discontinuation or reduction of the use of anesthetic agents in endoscopic procedures, may be a promising approach.^[23,24]^ It can be employed in a multitude of settings, including fracture-joint reduction in emergency departments; gynecological patients for acute pain, childbirth, pelvic examination, and abortion; and bone marrow aspiration, breast biopsy, dental interventions, and burn dressings.^[25,26]^ Additionally, there have been studies on the use of hypnosis in endoscopic interventions, however, there is a paucity of literature. This study represents a pioneering investigation into the potential benefits of hypnosis in ERCP.

The doses of midazolam and propofol administered to the subjects in group 1 were significantly lower than those administered to the subjects in group 2. A reduction in sedoanalgesia may facilitate awakening resulting in a higher Alderete score. Nevertheless, no significant differences were observed in the Alderete scores of patients in this study. However, it seems reasonable to posit that patients receiving a lower dose of medication are likely to experience fewer side effects due to the medication. Furthermore, no significant difference was identified in the satisfaction scale scores of patients following the procedure. This indicates that a similar level of comfort can be achieved with reduced dosage of sedoanalgesia in patients undergoing hypnosis.

In studies comparing hypnosis and control groups in cases of upper gastrointestinal endoscopy, an anxiety scale was employed in addition to the hemodynamic and satisfaction scales. No significant difference was established in hemodynamic and vital parameters, such as SpO_2_ and satisfaction scale scores. Nevertheless, although this difference was not statistically significant, the experimental group exhibited lower anxiety levels than the control group.^[19]^ In contrast, our study did not employ an anxiety scale. No significant differences were observed in the satisfaction scale scores between the 2 groups. The present study revealed that the oxygen saturation levels observed at both the 3-minute and 10-minute intervals were higher in the hypnosis group. This may indicate that the vital parameters of the patients exhibited superior progression during the procedure owing to the administration of a reduced dosage of anesthetic agents. In terms of vital parameters, patients who received hypnosis exhibited a reduction in adverse effects and demonstrated superior vital parameter performance throughout the procedure. However, the mean heart rate at the 5-minute mark was higher in the hypnosis group. It is hypothesized that this was because the sedation group was exposed to lower doses of the medication.

The present study was limited by the small number of patients included in each group and its single-center design. Also, we did not employ an anxiety scale in the methods in contrast to similar studies. We did not randomize the groups in terms of indication for ERCP procedure and age. It is a known fact that different age groups are more receptive to hypnosis. Placebo or sham hypnosis was not performed for ethical reasons, similar to previous studies. On the other hand, it is advantageous that all study patients underwent ERCP performed by the same endoscopist. Due to the fact that hypnotizability varies in society, a professional can be used on a routine basis for consultation, especially for outpatient surgical procedures in terms of a protocol just like anesthesiology, the potential economic benefit or cost-effectiveness remains to be confirmed. We hope that our results demonstrating the efficacy of clinical hypnosis may help to guide future studies with larger and randomized groups of participants in conjunction with minimally invasive surgical procedures.

## 5. Conclusions

The administration of hypnosis during ERCP in selected patients in experienced centers reduces the required dosage of anesthesia. The use of hypnosis can enhance the comfort and efficacy of the procedure, particularly in patients with a high number of comorbidities and in whom anesthesia is contraindicated. It is our contention that prospective, randomized studies utilizing hypnosis for anesthesia during ERCP in a larger patient sample are required.

## Author contributions

**Conceptualization:** Ufuk Arslan.

**Data curation:** Ufuk Arslan, Direnç Yiğit, Yiğit Düzköylü.

**Investigation:** Yiğit Düzköylü.

**Methodology:** Ufuk Arslan, Şeyda Efsun Özgünay.

**Project administration:** Şeyda Efsun Özgünay.

**Resources:** Yiğit Düzköylü.

**Software:** Direnç Yiğit.

**Supervision:** Ufuk Arslan.

**Validation:** Şeyda Efsun Özgünay, Direnç Yiğit.

**Writing – original draft:** Direnç Yiğit, Yiğit Düzköylü.

**Writing – review & editing:** Yiğit Düzköylü.
